# Thapsigargin-Stimulated LAD2 Human Mast Cell Line Is a Potent Cellular Adjuvant for the Maturation of Monocyte-Derived Dendritic Cells for Adoptive Cellular Immunotherapy

**DOI:** 10.3390/ijms22083978

**Published:** 2021-04-12

**Authors:** Pavla Taborska, Dmitry Stakheev, Jirina Bartunkova, Daniel Smrz

**Affiliations:** Department of Immunology, Second Faculty of Medicine, Charles University and University Hospital Motol, 15006 Prague, Czech Republic; Pavla.Taborska@fnmotol.cz (P.T.); dmitry.stakheev@lfmotol.cuni.cz (D.S.); jirina.bartunkova@lfmotol.cuni.cz (J.B.)

**Keywords:** LAD2 human mast cells, dendritic cells, maturation, adoptive cellular immunotherapy

## Abstract

The preparation of dendritic cells (DCs) for adoptive cellular immunotherapy (ACI) requires the maturation of ex vivo-produced immature(i) DCs. This maturation ensures that the antigen presentation triggers an immune response towards the antigen-expressing cells. Although there is a large number of maturation agents capable of inducing strong DC maturation, there is still only a very limited number of these agents approved for use in the production of DCs for ACI. In seeking novel DC maturation agents, we used differentially activated human mast cell (MC) line LAD2 as a cellular adjuvant to elicit or modulate the maturation of ex vivo-produced monocyte-derived iDCs. We found that co-culture of iDCs with differentially activated LAD2 MCs in serum-containing media significantly modulated polyinosinic:polycytidylic acid (poly I:C)-elicited DC maturation as determined through the surface expression of the maturation markers CD80, CD83, CD86, and human leukocyte antigen(HLA)-DR. Once iDCs were generated in serum-free conditions, they became refractory to the maturation with poly I:C, and the LAD2 MC modulatory potential was minimized. However, the maturation-refractory phenotype of the serum-free generated iDCs was largely overcome by co-culture with thapsigargin-stimulated LAD2 MCs. Our data suggest that differentially stimulated mast cells could be novel and highly potent cellular adjuvants for the maturation of DCs for ACI.

## 1. Introduction

Mast cells (MCs) are non-dividing, long-living, terminally differentiated, tissue- or mucosa-resident cells [1]. MCs are notoriously associated with asthma, allergy, atopy, and anaphylaxis [1,2]. MCs play a significant role in cancer [3]. However, since the current state of knowledge shows a deep controversy on MCs’ role in cancer, this role is still largely unknown [4]. The reason for this controversy is that the type of MC stimulation decides whether the response is pro-inflammatory or anti-inflammatory [5]. In vivo, MCs are exposed to a variety of stimuli, whose resultant impact on the regulation of the immune system is difficult to determine. However, an ex vivo stimulation of MCs can lead to a defined response, the consequence of which on individual components of the immune system could be determined. One of the immune cell types that MCs impact is dendritic cells (DCs). DCs were found to either directly interact with MCs [6,7] or become modulated by the biologically active compounds that the activated MCs produce after their activation [8,9,10].

Ex vivo-produced DCs are used for adoptive cellular immunotherapy (ACI) [11,12,13]. As their therapeutic use in immunotherapy is currently expanding, there is a need to improve their therapeutic performance. The DCs’ therapeutic performance depends on their phenotype, which depends on the source cells used for the DC production and the cell culture conditions [14,15]. For ACI, DCs are often produced by differentiation from peripheral blood monocytes, which leads to the production of immature DCs (iDCs). The iDCs are subsequently loaded with antigens and matured using a variety of maturation compounds [16,17]. The acquisition of the mature phenotype is critical for the efficient, professional presentation of the loaded antigens to allow DCs to trigger an efficient immune response against the cells expressing the antigen(s) [11].

In order to comply with the regulatory authorities and ensure the safety of therapeutic DCs, the production of DCs needs to be performed under strictly defined conditions that often prefer the cell to be cultured in serum-free media [18]. In addition, the use of the maturation compounds is also limited [18]. Combined, the serum-free conditions can lead to the production of DCs with distinct phenotypes [15], and the maturation may not be efficient.

In this study, we investigated whether a differentially stimulated and well-defined human mast cell line, LAD2 [19], could be used as a novel cellular adjuvant for the maturation of monocyte-derived DCs. The LAD2 MCs were stimulated with various stimuli and then co-cultured with iDCs in the absence or presence of the maturation compound polyinosinic:polycytidylic acid (poly I:C) [20,21]. The impact of the co-culture on DC maturation was determined in two types of monocyte-derived DCs that were differentially permissive to the poly I:C-mediated DC maturation.

## 2. Results

### 2.1. Differentially Stimulated LAD2 MCs Induce and Modulate DC Maturation

**Figure 1:**
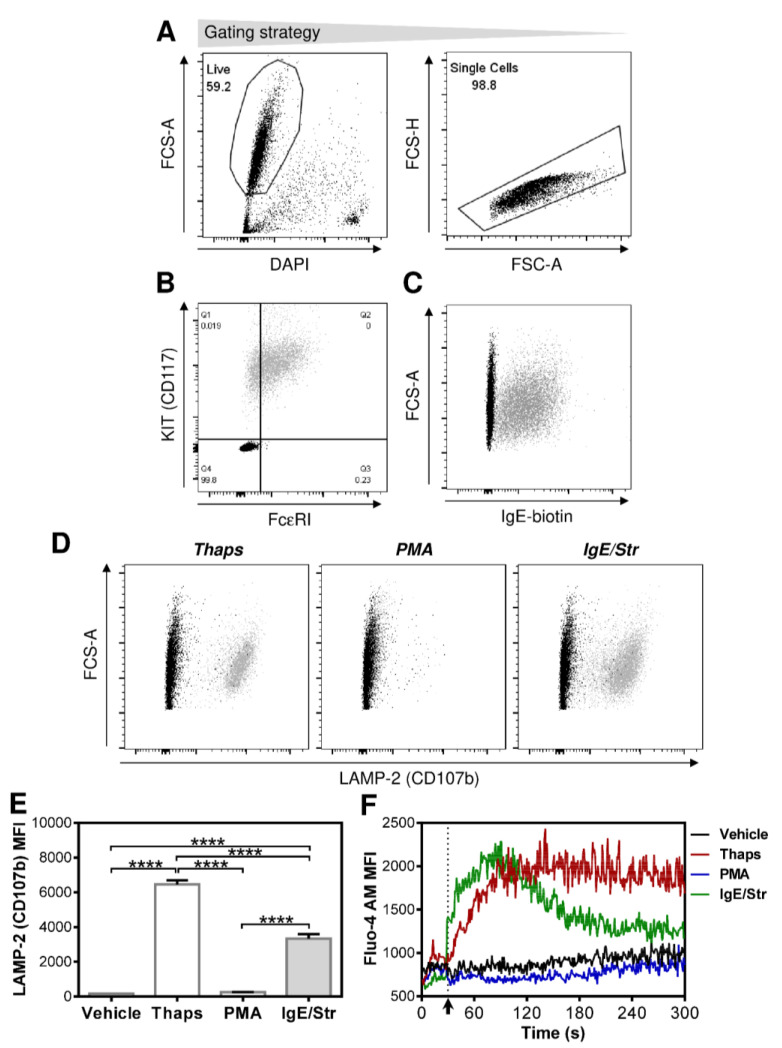
LAD2 mast cell (MC) phenotype and responses to differential stimulation. (**A**,**B**) LAD2 MCs were cultured without SCF for 18–24 h and then stained with fluorescently labeled CD117- and FcεRI-specific antibodies and analyzed by flow cytometry. The analyzed cells were gated (**A**) and analyzed for the expression of KIT receptor (CD117) and FcεRI. (**C**) LAD2 MCs were sensitized with biotinylated IgE for 18–24 h, and the bound IgE (IgE) was detected with fluorescently labeled streptavidin using flow cytometry. The gating strategy was as in (**A**). (**D**,**E**) The biotin-IgE-sensitized LAD2 MCs were stimulated with thapsigargin (Thaps, 1 µM), phorbol 12-myristate 13-acetate (PMA, 50 ng/mL), or streptavidin (IgE/Str, 10 µg/mL) for 30 min. The cell degranulation was determined by flow cytometry through the surface externalization of LAMP-2 (CD107b) (**D**). The gating strategy was as in (**A**), and the extent of degranulation in (**E**) was evaluated as the mean fluorescence intensities of the LAMP-2 (CD107b) staining (LAMP-2 (CD107b) MFI). (**F**) The IgE-biotin-sensitized LAD2 MCs were stained with Fluo-4 AM, rinsed, supplemented with DAPI (100 ng/mL; Thermo Scientific), and the baseline of the DAPI-negative cells’ Fluo-4 AM fluorescence was acquired for 30 s. Following the cells’ supplementation with the stimulant concentrations in (**D**,**E**), the DAPI-negative cells’ Fluo-4 AM fluorescence continued to be acquired for 4 min and 30 s. The kinetics of the acquired fluorescence (Fluo-4 AM MFI) was evaluated. The data in (**A**,**B**) and (**F**) are representative of at least 3 independent experiments. In (**F**), the arrow indicates the time of the cell stimulation. In (**E**), bars represent the mean of values and SEM determined in each group. The significance of differences among the group of cells is indicated (**** *p* < 0.0001, 1-way ANOVA with the Tukey post-test).

To investigate the impact of mast cells on the maturation of monocyte-derived DCs, we used human mast cell line LAD2 [19]. We first confirmed that LAD2 MCs expressed the KIT receptor (CD117) and the high-affinity receptor for IgE (FcεRI) on their surfaces (Figure 1A,B). To confirm that these cells respond to various stimuli, we sensitized the cells with biotinylated IgE (IgE-biotin) and confirmed its binding to the cell surface (Figure 1C). We then stimulated the sensitized cells with thapsigargin, an inhibitor of sarco/endoplasmic reticulum Ca2+-ATPases (SERCA) [22,23,24], phorbol 12-myristate 13-acetate (PMA), a structural analog of diacylglycerol and a specific activator of protein kinase C (PKC) [25], or by streptavidin-mediated crosslinking of FcεRI-bound IgE-biotin. Thapsigargin and FcεRI crosslinking led to LAD2 MC degranulation as determined by the surface externalization of LAMP-2 (CD107b) (Figure 1D,E). No degranulation was observed with PMA (Figure 1D,E). Based on previous reports [26,27,28] and the degranulation data, the following analyses also confirmed the expected kinetics of the calcium responses of differentially stimulated LAD2 MCs (Figure 1F). As shown, FcεRI crosslinking and thapsigargin induced the strong mobilization of intracellular calcium in LAD2 MCs. No calcium mobilization was observed after cell stimulation with PMA or vehicle alone (Figure 1F).

**Figure 2:**
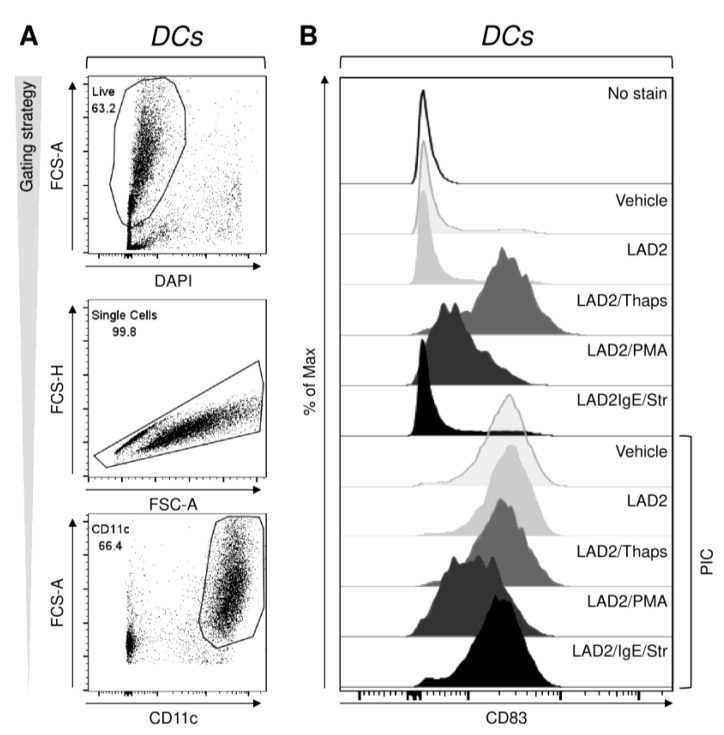
Impact of stimulated LAD2 MCs on the maturation of monocyte-derived dendritic cells (DCs) produced in the serum-containing medium. LAD2 MCs were, or were not (LAD2), stimulated with thapsigargin (LAD2/Thaps, 1 µM), PMA (LAD2/PMA, 50 ng/mL), or streptavidin (LAD2/IgE/Str, 10 µg/mL) for 30 min before the following co-culture with monocyte-derived immature(i)DCs. Monocyte-derived iDCs were produced in the serum-containing medium and matured for 20–24 h in F-bottom 96-well plate wells (0.2 × 10^6^ of iDCs in 200 µL of culture medium) in the presence or absence of poly I:C (PIC, 25 µg/mL) by no co-culture (Vehicle) or co-culture with differentially stimulated IgE-sensitized LAD2 MCs at a ratio 1:6 (LAD2 MCs:iDCs). The matured DCs were stained with the fluorescently labeled antibodies specific to the selected maturation markers and analyzed by flow cytometry. (**A**) The gating strategy for the analyses of the flow cytometry data to analyze DC maturation. (**B**) A histogram of the staining intensities of CD83 expression in DCs. The data (**A**,**B**) are representative of at least 4 donors, and the obtained data are evaluated in Figure 3.

**Figure 3:**
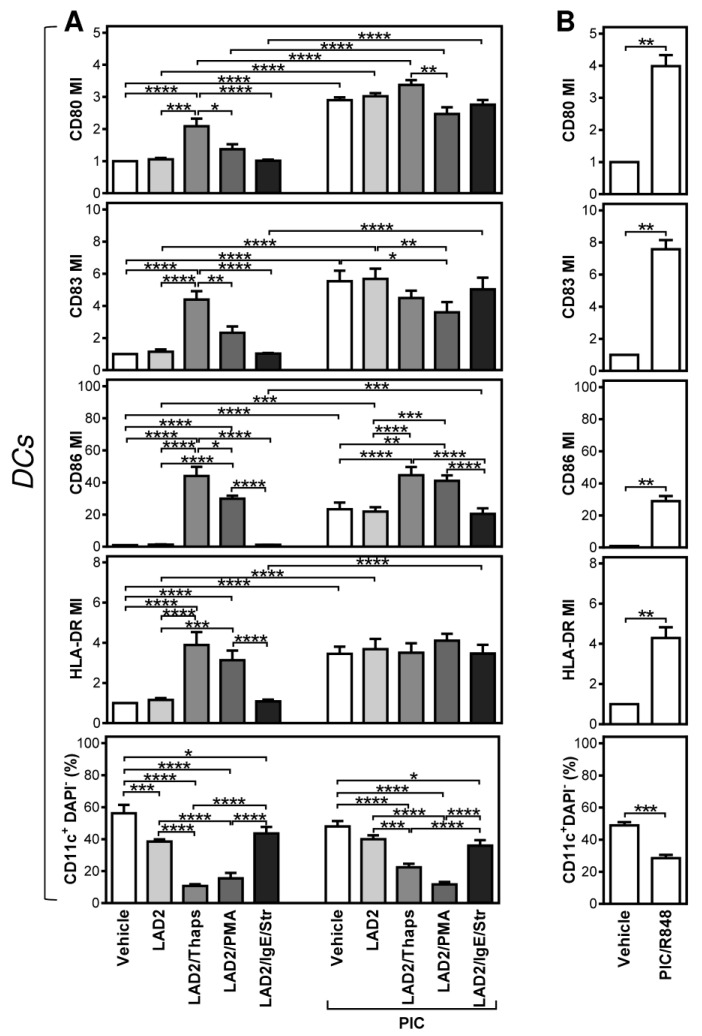
Evaluations of the impact of stimulated LAD2 MCs on the maturation of monocyte-derived DCs produced in the serum-containing medium. (**A**) The extent of DC maturation was evaluated through the maturation index (MI). The DC MI was calculated from the mean fluorescence intensities (MFIs) of the DC maturation marker staining in Figure 2 as the ratio between MFIs of the marker staining of the stimulated and non-stimulated (iDCs) DCs. The calculated DC MIs of individual samples were evaluated. The proportions of viable DCs (DAPI-CD11c+) were taken from Figure 2. (**B**) Monocyte-derived iDCs were produced in the serum-containing medium and matured for 20–24 h in F-bottom 96-well plate wells (0.2 × 10^6^ of iDCs in 200 µL of culture medium) in the presence or absence of poly I:C (PIC, 25 µg/mL) with R848 (10 µM). The DC MIs were calculated as in (**A**) and evaluated. In (**A**) and (**B**), bars represent the mean of values and SEM determined in each group. The significance of differences among the group of cells is indicated (* *p* < 0.05, ** *p* < 0.01, *** *p* < 0.001, **** *p* < 0.0001; (**A**): *n* = 4 donors, 1-way ANOVA with the Tukey post-test; (**B**): *n* = 4 donors, paired 2-tailed Student’s *t* test).

To see the impact of the differentially stimulated LAD2 MCs on DC maturation, we first stimulated IgE-biotin-sensitized LAD2 MCs with thapsigargin, PMA, or streptavidin. The differentially stimulated LAD2 MCs were then extensively rinsed with the culture media and co-cultured with immature monocyte-derived DCs at a ratio of 1:6 (LAD2 MCs:iDCs). These DCs were generated in the serum-containing medium, and the co-culture was performed in the presence or absence of the maturation compound, polyinosinic:polycytidylic acid (poly I:C), which is a TLR-3 agonist and is used for the maturation of DCs used for ACI [20,21,29,30]. The extent of DC maturation was determined through the surface expression of the DC maturation markers, CD80, CD83, CD86, and human leukocyte antigen(HLA)-DR [31]. As shown in Figure 2 and evaluated in Figure 3A, poly I:C enhanced the surface expression of all the tested maturation markers. No enhanced expression of the maturation markers was attained after co-culture with non-stimulated or FcεRI-stimulated LAD2 MCs. These cells also did not potentiate the poly I:C-induced DC maturation. However, the co-culture with either PMA- or thapsigargin-stimulated LAD2 MCs enhanced the expression of the tested maturation markers (Figure 3A). Whereas the PMA-stimulated LAD2 MCs enhanced expression of CD86 and HLA-DR, the thapsigargin-stimulated LAD2 MCs enhanced all the tested maturation markers to a comparable extent to poly I:C (Figure 3A). Poly I:C alone was less potent in enhancing the expression of CD86 than poly I:C combined with either thapsigargin- or PMA-stimulated LAD2 MCs (Figure 3A). However, the PMA-stimulated LAD2 MCs with poly I:C were, on the contrary, less effective than poly I:C alone in enhancing the expression of CD83 (Figure 3A).

One of the impacts co-culture of LAD2 MCs with iDCs delivered was the decrease in the content of viable DCs in the cell co-culture (Figure 3A, bottom panels). These data indicated that the co-culture was toxic to iDCs. However, we found that the same was also observed when iDCs were matured by the combination of poly I:C and R848, a synthetic TLR7/8 agonist often used alone or in combinations for DC maturation [16,32,33,34] (Figure 3B). These data suggested that the decreased viability of the matured DCs is rather a result of the exposure of iDCs to more complex maturation signals than to a LAD2 MC-elicited cytotoxic impact. The combined data showed that differentially stimulated LAD2 MCs could induce efficient DC maturation and differentially modulate DC maturation as elicited by other maturation compounds.

### 2.2. FcεRI-Stimulated LAD2 MCs Have Limited Potential to Promote Maturation of Maturation-Refractory iDCs

**Figure 4:**
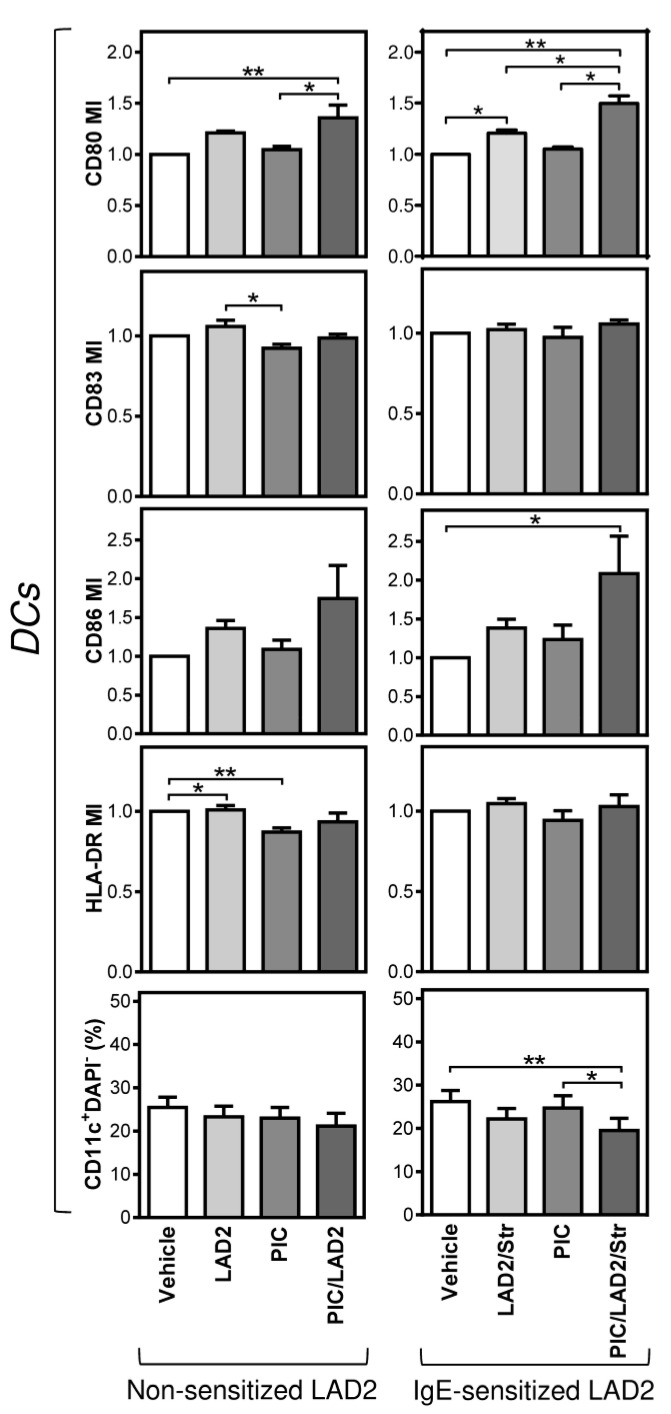
Evaluations of the impact of FcεRI-stimulated LAD2 MCs and poly I:C on the maturation of monocyte-derived DCs produced in the serum-free medium. LAD2 MCs were (right panels), or were not (left panels), sensitized with biotinylated IgE. Then, the cells were placed directly in the following co-culture with monocyte-derived immature(i)DCs with (LAD2/Str), or without (LAD2), streptavidin (Str, 10 µg/mL). Monocyte-derived iDCs were produced in the serum-free medium and matured for 20–24 h in F-bottom 96-well plate wells (0.2 × 10^6^ of iDCs in 200 µL of culture medium) in the presence or absence of poly I:C (PIC, 25 µg/mL) by no co-culture (Vehicle) or co-culture with LAD2 MCs at a ratio 1:6 (LAD2 MCs:iDCs). The matured DCs were stained with the fluorescently labeled antibodies specific to the selected maturation markers and analyzed by flow cytometry as in Figure 2. The maturation indexes (MIs) for individual markers and the proportions of viable DCs (DAPI-CD11c+) were evaluated. Bars represent the mean of values and SEM determined in each group. The significance of differences among the group of cells is indicated (* *p* < 0.05, ** *p* < 0.01, *** *p* < 0.001, **** *p* < 0.0001; *n*= 6 donors, 1-way ANOVA with the Tukey post-test).

The production of DCs for ACI prefers serum-free conditions [18,29,30], particularly due to the serum-elicited cell product’s variability [35,36]. However, the serum-free conditions often impact the phenotype of the generated DCs, including their capability to mature after exposure to maturation compounds [15]. In the next series of experiments, we investigated how LAD2 MCs impact the maturation of monocyte-derived DCs produced in the serum-free medium CellGro [21,37]. We found that, contrary to the serum-containing medium, iDCs generated in CellGro were refractory to maturation with poly I:C (Figure 4). This poly I:C maturation was only minimally, and only for some maturation markers, enhanced upon the co-culture of iDCs with LAD2 MCs (1:6 ratio, LAD2 MCs:iDCs) (Figure 4, left panels). Only slightly better results were obtained when the co-cultured LAD2 MCs were IgE-sensitized before the maturation and stimulated through FcεRI crosslinking during the co-culture. Under this setting, LAD2 MCs degranulated directly into the co-culture media. However, even under this setting, the impact on the poly I:C-induced maturation was limited as only two maturation markers, CD80 and CD86, showed a significant enhancement in their surface expression (Figure 4, right panels). Regardless, overall, these data showed that non-stimulated or FcεRI-stimulated LAD2 MCs were largely unable to efficiently overcome the maturation-refractory phenotype of monocyte-derived iDCs generated in the serum-free medium.

### 2.3. The Thapsigargin-Stimulated LAD2 MCs Induce Maturation in the Maturation-Refractory iDCs

**Figure 5:**
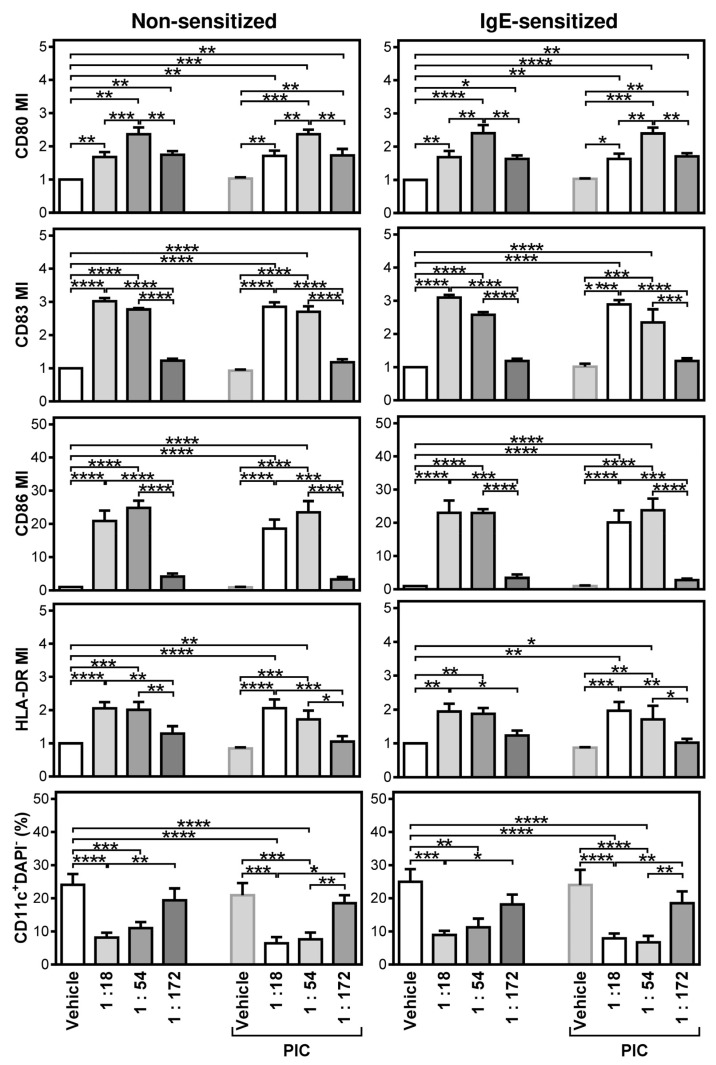
Evaluations of the impact of thapsigargin-stimulated LAD2 MCs and poly I:C on the maturation of monocyte-derived DCs produced in the serum-free medium. Monocyte-derived immature(i)DCs were produced in the serum-free medium and matured for 20–24 h in F-bottom 96-well plate wells (0.2 × 10^6^ of iDCs in 200 µL of culture medium) in the presence or absence of poly I:C (PIC, 25 µg/mL) by no co-culture (Vehicle) or co-culture with thapsigargin-stimulated LAD2 MCs at a ratio 1:18, 1:54, or 1:172 (LAD2 MCs:iDCs). The matured DCs were stained with the fluorescently labeled antibodies specific to the selected maturation markers and analyzed by flow cytometry as in Figure 2. The maturation indexes of DCs (MIs) for individual markers and the proportions of viable DCs (DAPI-CD11c+) were evaluated. Bars represent the mean of values and SEM determined in each group. The significance of differences among the group of cells is indicated (* *p* < 0.05, ** *p* < 0.01, *** *p* < 0.001, **** *p* < 0.0001; *n*= 6 donors, 1-way ANOVA with the Tukey post-test).

The serum-free conditions showed that iDCs can become refractory to their maturation with conventional maturation compounds and that this phenotype is also resilient to the modulation with non-stimulated or receptor-stimulated LAD2 MCs. We next investigated whether the non-specific stimulation of LAD2 MCs could break the maturation-refractory DC phenotype. We selected the thapsigargin stimulation for this series of experiments because it had previously shown the best performance with iDCs produced in the serum-containing medium. We also investigated the co-culture impact upon decreased ratios; the thapsigargin-stimulated LAD2 MCs were co-cultured with iDCs at 1:18, 1:54, or 1:172 (LAD2 MCs:iDCs) ratios. As shown in Figure 5 (left panels), the co-culture of iDCs with the thapsigargin-stimulated LAD2 MCs induced the strong maturation of DCs, as determined by the enhanced expression of all the tested maturation markers. This strong maturation was attained even at the co-culture ratio 1:54 (LAD2 MCs:iDCs). The expression of the CD80 maturation marker was attained even at the co-culture ratio 1:172 (LAD2 MCs:iDCs). The viability trend was comparable to that observed during the maturation in the serum-containing medium. The data also showed that poly I:C or IgE sensitization (Figure 5, right panels) did not contribute to the extent of DC maturation. Therefore, the maturation-refractory phenotype of iDCs was overcome solely by the thapsigargin-stimulated LAD2 MCs.

## 3. Discussion

This study showed the human mast cell line LAD2 as a new and highly potent cellular modulator of monocyte-derived DCs. The differentially stimulated LAD2 MCs were able to modulate poly I:C-mediated maturation of iDCs generated in a serum-containing medium. More importantly, however, the thapsigargin-stimulated LAD2 MCs overcame the maturation-refractory phenotype of iDCs generated under the serum-free condition.

MCs are immune cells of immense modulatory potential. Upon their stimulation, a large number of biologically active compounds are released [38,39]. Dysregulated MC function or activation of MCs is often the cause of many diseases, including asthma, allergy, atopy, or other mast cell activation-related disorders [1,2]. In tumors, these cells were found to foster immunosuppression [40] or decrease checkpoint immunotherapy efficacy [41]. On the other hand, MCs can be associated with a favorable prognosis [42,43]. However, the dividing line between MCs’ pro- or anti-tumorigenic properties in vivo is very slim [4]. In this study, we, however, showed that the MCs’ immense immunomodulatory potential could be used to prepare ex vivo-produced DCs for ACI. Our data showed that this immense modulatory potential could be elicited through the stimulated but not non-stimulated MCs. The data, however, showed that not every stimulation provided the desired maturation signals, as observed for the FcεRI-stimulated LAD2 MCs that were not inducing or promoting the DC maturation, although these cells were strongly degranulating before their co-culture with iDCs. The strong degranulation of stimulated LAD2 MCs was obviously not the measure of providing iDCs with the maturation signals because no degranulation was observed in PMA-stimulated LAD2 MCs and still the strong DC maturation or enhancement of the maturation with poly I:C followed during the subsequent co-culture.

The therapeutic performance of ex vivo-produced DCs for ACI largely depends on their maturation. Current protocols attempt to increase the maturation of DCs by multiple compounds, which often target the pattern recognition receptors, namely TLRs [44]. However, many of these, often highly potent, compounds, such as lipopolysaccharides (LPS), are not permitted for the maturation of DCs produced for clinical use, mainly due to endotoxin level regulation [45]. Their replacement for other compounds may sometimes provide a comparable maturation-inducing efficacy [46]. However, this efficacy could be compromised when the source cells for the DC production are adversely affected by the patient’s disease, or the DC production requires altered culture conditions in order to comply with the requirements for their clinical use. In this study, we demonstrated that two different culture conditions for DC production could lead to the production of iDCs with largely different properties. Whereas the serum-containing medium allowed the production of iDCs that were permissive to the poly I:C-mediated maturation, the serum-free medium led to the production of iDCs that were refractory to the poly I:C-mediated maturation. These data showed that the culture medium adjustment could not only impact the DC phenotype [15] but also compromise the permissiveness of iDCs to the maturation with a single DC maturation-inducing compound. This maturation-refractory phenotype was also largely resilient to the maturation settings where FcεRI-stimulated LAD2 MCs degranulated directly into the co-culture medium in the presence of poly I:C. These data showed that single immunoreceptor-stimulated MCs could not overcome the iDCs’ maturation-refractory phenotype.

Apart from other immune cell types, MCs also express various receptors [47,48] that sense the surrounding microenvironment for potentially dangerous compounds to respond to these compounds and mobilize the body’s protective response [49]. Among these compounds are venoms or chemical compounds [49]. Once a danger is sensed, MCs can produce massive amounts of compounds to neutralize the danger, including toxins such as snake or bee venoms [50]. This response may also counteract the current regulatory settings of the immune cells [51]. In this study, we indeed showed that this could truly happen. Short and non-toxic (data not shown) exposure of LAD2 MCs to thapsigargin, a compound which is otherwise toxic to cells after chronic exposure [52,53], caused not only the massive degranulation of LAD2 MCs [26] but the stimulated cells were able, after the extensive thapsigargin removal, to induce the strong maturation of the otherwise maturation-refractory iDCs. This finding was even more striking when the strong DC maturation was attained upon the co-culture with iDCs at a ratio of 1:54 (LAD2 MCs:iDCs), and even the ratio of 1:172 (LAD2 MCs:iDCs) was still able to enhance the expression of the maturation marker CD80 significantly—something that poly I:C was unable to attain in these maturation-refractory iDCs.

The mechanism through which thapsigargin-stimulated LAD2 MCs were able to induce efficient DC maturation during co-culture is unknown. Short exposure of LAD2 MCs to thapsigargin induces their degranulation and cytokine production [27,28]. Although the released granules contain many biologically active products [38,39] known to promote DC maturation [54], and the stimulation also induces de novo cytokine production [28], a similar response is also induced in the immunoreceptor (FcεRI)-stimulated LAD2 MCs [26,28]. However, this immunoreceptor (FcεRI)-mediated stimulation was inefficient in lending LAD2 MCs the ability to induce DC maturation. This indicates that thapsigargin must impact LAD2 MCs in another way that provides the cells with the exceptional ability to efficiently mature DCs. This impact is presumably not driven by the extent of LAD2 MC activation because IgE sensitization, which is known to enhance MCs’ effector responses [55,56], did not notably contribute to the extent of the DC maturation. This impact, however, could be associated with sarcoplasmic/endoplasmic reticulum (ER) stress. A recent study has shown that short and non-toxic treatment of epithelial cells with thapsigargin triggers ER stress, which appears to be responsible for inducing a spectrum of host antiviral defenses [57]. This thapsigargin-elicited ER stress was also associated with the induction of heat-shock proteins known to promote DC maturation [58]. However, whether ER stress is the driver of the LAD2 MC-mediated maturation of DCs remains to be elucidated. Regardless, the results of the study demonstrated that thapsigargin-stimulated LAD2 MCs are a highly potent maturation agent capable of breaking the maturation-refractory phenotype of ex vivo-produced, monocyte-derived iDCs. Therefore, these findings show that MCs’ immense immunomodulatory powers can be ex vivo harnessed for efficient preparation of DCs for ACI.

## 4. Materials and Methods

### 4.1. Specimens

LAD2 MCs [19] were obtained under the Material Transfer Agreement MTA (no. 2012-2809) from NIAID/NIH (Bethesda, MD, USA). The buffy coats were obtained from the Institute of Hematology and Blood Transfusion in Prague. The donors signed written informed consent to participate in the study, and all experimental protocols were approved by the institutional research committee—the Ethics Committee of the University Hospital Motol in Prague. The experiments were performed in accordance with the 1964 Helsinki declaration and its later amendments or comparable ethical standards.

### 4.2. LAD2 MC Stimulation and Analyses

LAD2 MCs were cultured in serum-free culture medium (SP medium; StemPro-34 culture medium with a supplement (Thermo Scientific, Waltham, MA, USA), 100 U/mL penicillin–streptomycin, 2 mM GlutaMax (Thermo Scientific)) supplemented with 100 ng/mL of human stem cell factor (SCF, PeproTech, Rocky Hill, NJ, USA). The cells (0.5–1.0 × 10^6^ cells/mL) were, or were not, sensitized with biotinylated IgE (200 ng/mL; product# BPD-DIA-HE1B-01, Enzo Life Sciences, Farmingdale, NY, USA) for 18–24 h at 37 °C and 5% CO_2_. The cells were harvested, twice rinsed with 5 mL SP medium without SCF, and stimulated with thapsigargin (1 µM; Sigma-Aldrich, St. Louis, MO, USA) [26,28], PMA (50 ng/mL; Sigma-Aldrich) or streptavidin (10 µg/mL; Sigma-Aldrich) for 30 min at 37 °C and 5% CO_2_. The stimulated cells were harvested and three-times rinsed with 5 mL of the corresponding DC culture medium in which the maturation was performed. The cells were then resuspended in the DC culture media at desired concentrations.

To analyze the IgE binding to LAD2 MCs, the cells (0.5–1.0 × 10^6^ cells/mL) were sensitized with the biotinylated IgE as above. To analyze the expression of CD117 and FcεRI on the surfaces of LAD2 MCs, the cells were starved of SCF for 18–24 h at 37 °C and 5% CO_2_. The IgE-sensitized or SCF-starved LAD2 MCs were transferred to V-bottom 96-well plates (Nalgene, Rochester, NY, USA), stained with avidin-FITC (Becton Dickinson, Franklin Lakes, NJ, USA) or specific antibodies, CD117-APC and FcεRI-FITC (Becton Dickinson). The cells were washed with ice-cold PBS containing 2 mM EDTA (PBS/EDTA), resuspended in ice-cold PBS/EDTA with DAPI (100 ng/mL; Thermo Scientific), and analyzed by a FACSAria II (Becton Dickinson). The flow cytometry data were analyzed by FlowJo software (Tree Star, Ashland, OR, USA). The degranulation of stimulated LAD2 MCs was determined through LAMP-2 (CD107b) externalization assay as previously described [59]. The calcium response kinetics of stimulated LAD2 MCs were determined with minor modifications by flow cytometry as described previously [27]. Briefly, the IgE-biotin-sensitized LAD2 MCs were harvested, pelleted, and resuspended in HEPES/BSA buffer [27] containing Fluo-4 AM (9.12 µM (10 µg/mL); Thermo Scientific) and sulfinpyrazone (0.3 mM [60]; Sigma-Aldrich). The cells were incubated at 37 °C and 5% CO_2_ for 30–45 min, pelleted, and rinsed with HEPES/BSA buffer with sulfinpyrazone and without Fluo-4 AM. The cells were resuspended in HEPES/BSA/sulfinpyrazone buffer, incubated at 37 °C for over 5 min, supplemented with DAPI (100 ng/mL; Thermo Scientific), and immediately analyzed by flow cytometry using FACSAria II (Becton Dickinson). The baseline of the DAPI-negative cells’ Fluo-4 AM fluorescence was acquired in the 37 °C-chamber for 30 s, and, following the cells’ supplementation with the indicated stimulant’s concentration, the DAPI-negative cells’ Fluo-4 AM fluorescence continued to be acquired in the 37 °C chamber for 4 min and 30 s. The acquired DAPI-negative cells’ Fluo-4 AM fluorescence kinetics were evaluated by FlowJo software (Tree Star, Ashland, OR, USA).

### 4.3. Preparation of iDCs

Peripheral blood mononuclear cells (PBMCs) were isolated from buffy coats by density gradient as described previously [61]. For iDC preparation in the serum-containing medium, the gradient-isolated PBMCs were fractioned into adherent and nonadherent cells as described previously [59]. The monocytes (adherent fraction) were differentiated into iDCs using fetal bovine serum-containing culture medium (KM medium; RPMI 1640 medium (Thermo Scientific), 10% heat-inactivated fetal bovine serum (HyClone, GE Healthcare Life Sciences, South Logan, UT, USA), 100 U/mL penicillin–streptomycin, 2 mM GlutaMax (Thermo Scientific)) supplemented with 1000 IU/mL of granulocyte-macrophage colony-stimulating factor (GM-CSF) and 1000 IU/mL of IL-4 (Immunotools, Friesoythe, Germany). The cells were cultured as described [61]. For iDC preparation in the serum-free medium, the PBMC fractionation and monocyte differentiation into iDCs were performed in CellGro medium (CellGenix, Freiburg, Germany) supplemented with 2 mM GlutaMax, 100 U/mL penicillin–streptomycin, and the above-indicated cytokines. The cells were cultured as described [61].

### 4.4. DC Maturation and Analyses

The produced iDCs were harvested, pelleted at 240× *g* for 10 min at RT, and resuspended in fresh KM or CellGro medium with GM-CSF and IL-4 (1000 IU/mL; Immunotools) at a concentration of 2 × 10^6^ cells/mL. The cell suspension (100 µL; 0.2 × 10^6^ iDCs) was transferred to F-bottom 96-well plate wells with 100 µL of the above corresponding KM or CellGro medium containing, or not, non-stimulated or differentially stimulated LAD2 MCs. The cells were, or were not, then supplemented with poly I:C (25 µg/mL; Hycult Biotech, Uden, Netherland). Alternatively, the cells were also supplemented with R848 (10 µg/mL; Enzo Life Sciences, Farmingdale, NY, USA). The cell co-culture was then extensively resuspended and cultured for 18–24 h (37 °C, 5% CO_2_).

For determination of DC maturation, the co-cultured cells were transferred to a V-bottom 96-well plate (Nalgene) and stained as above (Section 4.2) with the following antibodies: CD11c-APC (Exbio, Prague, Czech Republic) in combination with CD80-FITC, CD86-PE, CD83-FITC (Beckman Coulter, Brea, CA, USA), or HLA-DR-Pe-Cy7 (Becton Dickinson) for 30–60 min at 4 °C. The cells were rinsed with ice-cold PBS/EDTA and analyzed by flow cytometry as above (Section 4.2). The extent of the DC maturation for each maturation marker was determined as the maturation index (MI), which expresses the relative difference between the expression of a DC maturation marker before and after DC maturation. The MI was calculated as the ratio between the mean fluorescence intensities (MFIs) of the marker staining of the stimulated (matured) and non-stimulated (iDCs) DCs.

### 4.5. Statistical Analysis

The means and SEM were calculated by GraphPad Prism 6 (GraphPad software, La Jolla, CA, USA) from the indicated sample size (*n*). Statistical significance (* *p* < 0.05, ** *p* < 0.01, *** *p* < 0.001, **** *p* < 0.0001) was determined by the indicated test.

## 5. Conclusions

LAD2 MCs stimulated with thapsigargin are a novel and highly potent maturation agent of ex vivo-produced, monocyte-derived DCs for ACIs. Our data suggest that selectively stimulated MCs could be highly potent cellular adjuvants for the maturation of DCs for ACI.

## Abbreviations

MC: mast cell, DC: dendritic cell, ACI: adoptive cellular immunotherapy, iDC: immature DC, poly I:C: polyinosinic-polycytidylic acid, FcεRI: high-affinity receptor for IgE, IgE-biotin: biotinylated IgE, PMA: phorbol 12-myristate 13-acetate, HLA-DR: human leukocyte antigen DR, ER: sarcoplasmic/endoplasmic reticulum.
